# Seroprevalence of *Toxoplasma gondii *infection in household and stray cats in Lanzhou, northwest China

**DOI:** 10.1186/1756-3305-4-214

**Published:** 2011-11-09

**Authors:** Song-Ming Wu, Xing-Quan Zhu, Dong-Hui Zhou, Bao-Quan Fu, Jia Chen, Jian-Fa Yang, Hui-Qun Song, Ya-Biao Weng, De-He Ye

**Affiliations:** 1 State Key Laboratory of Veterinary Etiological Biology, Key Laboratory of Veterinary Parasitology of Gansu Province, Lanzhou Veterinary Research Institute, Chinese Academy of Agricultural Sciences, Lanzhou, Gansu Province 730046, PR China; 2 College of Veterinary Medicine, South China Agricultural University, Guangzhou, Guangdong Province 510642, P R China; 3 College of Animal Science and Technology, Yunnan Agricultural University, Kunming, Yunnan Province 650201, PR China; 4 College of Veterinary Medicine, Gansu Agricultural University, Lanzhou, Gansu Province 730070, PR China

## Abstract

**Background:**

*Toxoplasma gondii *is an important protozoan parasite infecting humans and almost all warm-blooded animals. As the only definitive host, cats play a crucial role in the transmission of *T. gondii *infection by shedding parasite oocysts in their feces. However, little information on *T. gondii *infection in cats was available in Lanzhou, northwest China. This study was performed to determine the seroprevalence of *T. gondii *infection in household and stray cats in Lanzhou, northwest China.

**Results:**

A total of 221 (179 households and 42 strays) blood samples were collected from clinically healthy cats admitted to several pet hospitals located in Lanzhou City, between November 2010 and July 2011 for the serological detection of *T. gondii *infection. The majority (207) of these cats represented Chinese Lihua cats. 47 of 221 (21.3%) examined cats were seropositive for *T. gondii *infection using the modified agglutination test (MAT) at the cut-off of 1:25. The seroprevalence in household and stray cats was assessed to be 15.6% and 45.2%, respectively, and the difference was statistically significant (*P <*0.05). The seroprevalence ranged from 15.1% to 25.8% among different age groups, but the differences were not statistically significant (*P >*0.05). Studies showed that there was no relationship between seroprevalence and the gender (*P >*0.05).

**Conclusions:**

The present survey indicated the high seroprevalence of *T. gondii *in cats in Lanzhou, northwest China, which poses a threat to animal and human health. Therefore, measures should be taken to control and prevent toxoplasmosis of cats in this area.

## Background

*Toxoplasma gondii *is an obligate intracellular parasite, affecting humans and a wide range of warm-blooded animals worldwide [1-3]. *T. gondii *infection is a global concern, and about one third of the human population has been exposed to this parasite [1]. Toxoplasmosis is one of the most important food-borne diseases that can cause toxoplasmic encephalitis in immuno-compromised patients, blindness, abortion, fetal abnormalities or even prenatal death in congenital cases [3,4]. Humans or animals can acquire *T. gondii *infection post-natally by ingestion of undercooked or raw meat from infected animals, or ingestion of food or water contaminated with oocysts excreted by infected felids, or ingestion of oocysts from the environment by accident [1,5].

Felids are considered the only definitive hosts of *T. gondii *playing a crucial role in the transmission of the parasite [6]. Cats infected by *T. gondii *may pose a potential threat to public health, because they can shed and excrete environmentally resistant oocysts in their feces [7,8]. Household cats are one of the most intimate companions of humans. By frequent contact with cats, people may increase their risk of acquiring *T. gondii *infection. More importantly, stray cats usually wander everywhere and play a more important role in the transmission of toxoplasmosis to other animals and humans [6].

Surveys of *T. gondii *infection in stray and household cats have been reported extensively in the world [1,9], including mainland China [10-13]. However, little is known about the infection of *T. gondii *in stray and household cats in Lanzhou, northwest China. The objective of the present study was to determine the seroprevalence of *T. gondii *infection in stray and household cats in Lanzhou.

## Materials and methods

### The investigated city

The survey was conducted in Lanzhou City (35°5"~38° N, 102°30"~104°30" E), which is the capital of Gansu province, covering an area of approximately 13, 000 square meters in northwest China. This city is at an elevation of approximately 1, 500 meters, crossed by the Yellow River from west to east, having a characteristic ribbon basin geography. The climate is temperate and continental monsoonal with an average annual temperature of 9.3°C and annual precipitation of 360 mm.

### Naturally infected cats

Between November 2010 and July 2011, a total of 221 blood samples were obtained from stray and household cats in Lanzhou. These clinically healthy cats were admitted into pet hospitals located in three districts of Lanzhou City, namely Chengguan District, Anning District and Xigu District, for the serological detection of *T. gondii *infection. Information regarding the breed, age, gender and geographical origin of pet cats were obtained from their owners, and the biometric data of stray cats were estimated based on body condition and dental age. Blood samples were kept at room temperature for 2 h, centrifuged at 3, 000 rpm for 5 min, and the separated serum samples were stored at -20°C until further analysis. This study was approved by the Animal Ethics Committee of Lanzhou Veterinary Research Institute, Chinese Academy of Agricultural Sciences.

### Serological examination

Antibodies to *T. gondii *were determined in cat sera by the modified agglutination test (MAT) as described previously [14]. In this study, we chose MAT because it is sensitive and specific for detecting *T. gondii *antibodies in many animals as compared to other serologic methods [15-17]. In brief, sera were added to "U" bottom 96 well microtiter plates, diluted two-fold starting from 1:25 to 1:1600, the plates were shaken for 2 min and then incubated at 37°C overnight without shaking. Sera with MAT titers of 1:25 or higher were considered positive, and those sera with dubious results were re-tested. Positive and negative controls were incorporated in each test.

### Statistical analysis

Differences in the seroprevalence of *T. gondii *infection between male and female cats, and among different age groups were analyzed using a Chi square test using the SPSS for Windows (Release 18.0 standard version, SPSS Inc., Chicago, Illinois). The differences were considered statistically significant when *P *< 0.05.

## Results

In this study, serum samples were obtained from a total of 211 clinically healthy cats (179 households and 42 strays). The majority (207) of these cats represented Chinese Lihua cats. 47 of 221 (21.3%) examined cats were seropositive for *T. gondii *infection by MAT at the cut-off of 1:25 (Table 1). Seroprevalence of *T. gondii *infection in household and stray cats were 15.6% and 45.2%, respectively. Among different age groups, the seroprevalence varied from 15.1% to 25.8% (Table 1).

**Table 1:** Seroprevalence of *Toxoplasma gondii *infection in household and stray cats by gender and age in Lanzhou, northwest China using modified agglutination test (MAT)

Cat groups	Types of cat						Total		
				
	Household cat			Stray cat					
	
	No. tested	No. positive	Prevalence (%)	No. tested	No. positive	Prevalence (%)	No. tested	No. positive	Prevalence (%)
Gender									
Male	87	17	19.5	17	9	52.9	104	26	25
Female	92	11	12	25	10	40	117	21	17.9
Age (years)									
< 1	41	4	9.8	12	4	33.3	53	8	15.1
2	65	12	18.5	24	11	45.8	89	23	25.8
3	37	6	16.2	3	2	66.7	40	8	20
≥3	36	6	16.7	3	2	66.7	39	8	20.5
Total	179	28	15.6	42	19	45.2	221	47	21.3

The seroprevalence in male cats was 25%, and in females it was 17.9% (Table 1). Table 2 shows the distribution of antibodies to *T. gondii *in household and stray cats determined using MAT, and antibody titers ranged from 1:25 to 1:1600 or higher. Table 3 shows the seroprevalence of *T. gondii *infection in household and stray cats in relation to their breeds.

**Table 2:** Antibody titers to *Toxopalsma gondii *infection in household and stray cats in Lanzhou, northwest China by modified agglutination test (MAT)

Types of cat	No. of sera with MAT titers of							
	
	1:25	1:50	1:100	1:200	1:400	1:800	≥1:1600	Total
Household cat	3	5	5	1	1	2	11	28
Stray cat	1	3	1	1	1	3	9	19
Total	4	8	6	2	2	5	20	47

**Table 3:** Seroprevalence of *Toxoplasma gondii *infection in cats by breed in Lanzhou, northwest China using modified agglutination test (MAT)

Breeds of cat	No. examined	No. positive	Prevalence (%)
Chinese Lihua cat	207	45	21.7
Chausie cat	1	0	0
Persian cat	5	0	0
Russian Blue cat	1	1	100
Dragen-Li cat	2	0	0
British Shorthair cat	2	1	50
Mainecoon cat	1	0	0
Highland Scottish Fold cat	2	0	0
Total	221	47	21.3

## Discussion

The overall prevalence of *T. gondii *infection in cats in Lanzhou was 21.3%, which was lower than that reported in some other countries, such as in Iran (32.1%) [1,18], lower than that observed in Guangzhou City (25.2%) [11], but higher than that in Zhengzhou City (15.5%) and Beijing City (14.1%) in China [19,20]. The differences in seroprevalences of *T. gondii *in cats are probably due to differences in ecological and geographical factors, serologic tests used and the living conditions for cats. In general, *T. gondii *oocysts are more likely to survive in warm and humid environments [1]. The warm and humid climate in southern China (such as Guangzhou) is favorable for the transmission of *T. gondii*, whereas the cold and dry climate in winter in Lanzhou may be less favorable for the spread of *T. gondii*.

The results indicated that prevalence of antibodies varied with ages, and *T. gondii *seroprevalence in older animals was generally higher than that in young animals, however, the differences were not statistically significant (*P >*0.05). Female cats had lower prevalence than the male animals, although the difference was not significant (*P >*0.05).

In this study, seroprevalence of *T. gondii *infection in stray cats was 45.2%, which is significantly higher than that (15.6%) in household cats (*P <*0.05), consistent with reports from some other countries [21,22]. Differences in their hunting habits, living conditions and animal welfare may attribute to the difference in *T. gondii *seroprevalence between household cats and stray cats. Table 2 shows that 20 (42.6%) of the 47 infected cats had anti-*T. gondii *titers of 1:1600 or higher, indicating that these cats suffered from severe infection and/or repeated exposure to *T. gondii*, shedding oocysts to the environment.

The present study examined seroprevalence of *T. gondii *infection in clinically healthy household and stray cats. Further investigations of *T. gondii *seroprevalence in diseased cats concurrently infected with other feline pathogens are warranted because previous studies have not provided critical evidence to demonstrate association between *T. gondii *and other feline pathogens [1].

## Conclusions

The results of the present survey revealed a high seroprevalence of *T. gondii *infection in cats in Lanzhou, especially in stray cats. Knowing that cats play an important role in the transmission of *T. gondii*, revealing thus a significant public health concern, integrated strategies with efficient management measures should be taken to prevent and control *T. gondii *infection in cats in this city.

## Competing interests

The authors declare that they have no competing interests.

## Authors' contributions

XQZ and DHY conceived and designed the study, and critically revised the manuscript. SMW, DHZ and JC performed the experiments, analysed the data and drafted the manuscript. BQF, JFY, HQS and YBW helped in study design, study implementation and manuscript revision. All authors read and approved the final manuscript.
